# A late eating midpoint is associated with increased risk of diabetic kidney disease: a cross-sectional study based on NHANES 2013–2020

**DOI:** 10.1186/s12937-024-00939-z

**Published:** 2024-03-23

**Authors:** Chun-feng Lu, Xiao-min Cang, Wang-shu Liu, Li-hua Wang, Hai-yan Huang, Xue-qin Wang, Li-hua Zhao, Feng Xu

**Affiliations:** 1 Department of Endocrinology, Affiliated Hospital 2 of Nantong University and First People’s Hospital of Nantong City, No. 666 Shengli Road, Nantong, 226001 China; 2 Department of Nursing, Affiliated Hospital 2 of Nantong University and First People’s Hospital of Nantong City, No. 666 Shengli Road, Nantong, 226001 China

**Keywords:** Eating midpoint, Eating duration, Diabetic kidney disease, NHANES

## Abstract

**Background:**

Modifying diet is crucial for diabetes and complication management. Numerous studies have shown that adjusting eating habits to align with the circadian rhythm may positively affect metabolic health. However, eating midpoint, eating duration, and their associations with diabetic kidney disease (DKD) are poorly understood.

**Methods:**

The National Health and Nutrition Examination Survey (2013–2020) was examined for information on diabetes and dietary habits. From the beginning and ending times of each meal, we calculated the eating midpoint and eating duration. Urinary albumin-to-creatinine ratio (UACR) ≥ 30 mg/g and/or estimated glomerular filtration rate (eGFR) < 60 mL/min/1.73 m^2^ were the specific diagnostic criteria for DKD.

**Results:**

In total, details of 2194 subjects with diabetes were collected for analysis. The overall population were divided into four subgroups based on the eating midpoint quartiles. The prevalence of DKD varied noticeably (*P* = 0.037) across the four categories. When comparing subjects in the second and fourth quartiles of eating midpoint to those in the first one, the odds ratios (ORs) of DKD were 1.31 (95% CI, 1.03 to 1.67) and 1.33 (95% CI, 1.05 to 1.70), respectively. And after controlling for potential confounders, the corresponding ORs of DKD in the second and fourth quartiles were 1.42 (95% CI, 1.07 to 1.90) and 1.39 (95% CI, 1.04 to 1.85), respectively.

**Conclusions:**

A strong correlation was found between an earlier eating midpoint and a reduced incidence of DKD. Eating early in the day may potentially improve renal outcomes in patients with diabetes.

**Supplementary Information:**

The online version contains supplementary material available at 10.1186/s12937-024-00939-z.

## Introduction

Diabetic kidney disease (DKD) is a prevalent microvascular consequence of diabetes, with its prevalence rising in response to the global rise in diabetes incidence [1]. The adverse effects of DKD extend beyond the heightened risk of advancing to end-stage renal disease [2]. They also substantially elevate the occurrence of cardiovascular disease and death [3], posing a severe concern to public health. Moreover, despite efforts to manage several risk factors associated with DKD, its development and advancement may still occur in some individuals with diabetes [4]. Hence, it has immense therapeutic importance to undertake any endeavor aimed at improving renal damage in diabetic individuals.

Circadian rhythms regulate physiological and behavioral rhythms in a nearly 24-hour cycle. The central clock of the hypothalamus located in the suprachiasmatic nucleus (SCN) mainly receives light stimulation, and then down-regulates the circadian rhythms of multiple organ systems to coordinate physiological processes [5]. Furthermore, apart from the SCN, circadian rhythm genes are also expressed in several additional brain areas and peripheral organs. It is worth mentioning that the expression of circadian rhythm genes in the kidney is ranked second only to that seen in the liver, indicating the significant regulatory function of circadian rhythm in kidney cells [6]. Peripheral clocks can become disengaged from the regulation of the SCN as a consequence of alterations in eating behaviors. Research has shown that deviations from the typical meal cycle may lead to disturbances in the circadian rhythm [7]. Numerous studies have shown that persistent disruptions in circadian rhythm have been implicated in the development of obesity, hypertension, insulin resistance, inflammation, aberrant glucose and lipid metabolism, among other conditions [8–10]. These characteristics are well recognized as significant risk factors for DKD [8–10]. Subsequently, the regulation of nutrition may be advantageous in mitigating the risk of DKD via the modulation of circadian rhythm.

Dietary modification, an essential element of diabetes therapy and its associated problems, involves the strategic adjustment of nutritional consumption [11]. Caloric restriction (CR) is a common dietary modification, and has the potential to mitigate several chronic illnesses, including cancer, diabetes, and chronic kidney disease (CKD) [12]. However, the increased hunger caused by CR and low adherence limit the clinical use of CR, resulting in focusing on searching for other dietary modifications. Observational and interventional studies indicate that the timing of meals is essential for maintaining health. Two observational studies showed that the delay in the first meal was associated with increased blood pressure, C-reactive protein (CRP) level, glucose and insulin levels, and decreased high-density lipoprotein cholesterol (HDL-C) level [13, 14]. Wieth et al. also observed that a later last meal of the day was associated with a higher level of glycosylated hemoglobin (HbA1c) [14]. Likewise, a large prospective cohort study revealed that later times of first and last meals significantly contribute to increased risks of cardiovascular disease [15]. Time restricted eating (TRE) is a novel dietary strategy that seeks to maintain a resilient circadian rhythm by limiting the duration of eating to a relatively brief timeframe without imposing restrictions on the nutritional quality and caloric content of the consumed foods [16]. The efficacy of TRE in enhancing metabolic health has been shown by its ability to decrease body weight, enhance insulin resistance, and regulate metabolism [17, 18]. However, Sutton et al. compared the results of previous TRE clinical studies and concluded that the effects of TRE interventions might depend on the specific timing of the eating window throughout the day [19]. Based on this, Sutton and his colleagues proposed early time-restricted eating (eTRE), specifically, subjects receiving eTRF intervention needed to finish eating before 3 PM [19]. In their study, a 5-week intervention of eTRE in male individuals with prediabetes resulted in substantial improvements in insulin levels, insulin sensitivity, blood pressure, and oxidative stress levels [19]. Notably, these improvements were seen without any notable changes in the participants’ body mass. Similarly, a cross-sectional research using data from the National Health and Nutrition Examination Survey (NHANES) showed that consuming meals later in the day was associated with a notable rise in the likelihood of having raised fasting glucose levels [20]. However, the duration of eating window did not have a significant impact on this risk [20]. Hence, it was hypothesized that eating early in the day in individuals diagnosed with diabetes might potentially provide kidney protective benefits.

This observational research sought to investigate the potential correlation between eating midpoint, eating duration, and the incidence of DKD using data obtained from the NHANES.

## Materials & methods

### Study population

**Fig. 1:**
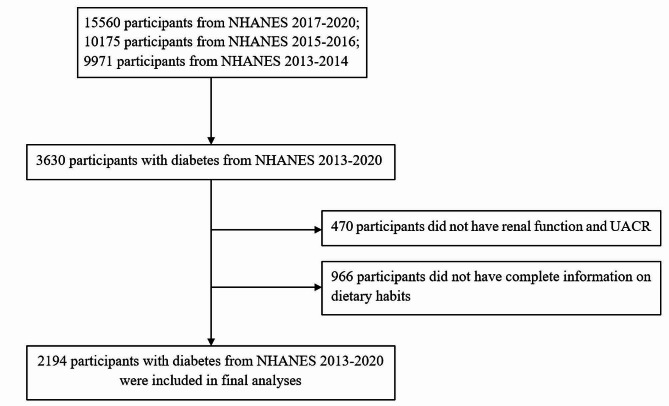
Flow chart of study population selection

The NHANES was undertaken by the Centers for Disease Control and Prevention with the aim of gathering comprehensive data on the nutritional and health status of both children and adults residing in the United States. The NHANES study used a sampling method that adhered to the principles of multi-stage, complicated, and probabilistic sampling to get a nationally representative sample [21]. The present study used the whole dataset including four cycles of the NHANES spanning the years 2013 to 2020. This research included individuals who were of adult age and diagnosed with diabetes. Diabetes was operationally defined as the presence of a documented medical history of diabetes and/or a HbA1c level equal to or over 6.5%. Subjects who had missing data about meal times, calorie intake, renal function, and urine albumin-to-creatinine ratio (UACR) were eliminated from the study. In addition, those who were following an extremely low-calorie diet were also eliminated from the study. Ultimately, the research included 2194 individuals. The detailed criteria of study population selection were seen in Fig. 1.

### Eating parameters

During each cycle, two 24-hour dietary recalls were administered to gather data pertaining to eating patterns, including meal timing and composition, and energy intake. The first dietary memory interviews were conducted by skilled dietary interviewers, followed by a subsequent dietary recall gathered by telephone after a time interval ranging from 3 to 10 days. The parameters obtained included the initial and final meal times, as well as the calorie consumption. The eating duration was determined by measuring the time gap between the first and final meals, with the first meal being considered to have taken place after 5 AM. A five-step methodology was employed to measure the consumption of food and beverages throughout a 24-hour period. The term “meal” was operationally defined as a dietary consumption event that resulted in an energy intake above 1 kcal within a certain time frame. The eating midpoint was operationally defined as the midpoint between the eating window [20]. This was mathematically represented by the formula (eating duration/2) + first meal time [20]. The average of each metric was calculated based on the data obtained from the two 24-hour dietary recalls.

### Clinical variables

The demographic information obtained from the NHANES data included age, race, gender, family income, alcohol intake, smoking status, and body mass index (BMI).

### Laboratory variables

The detailed measurement method of renal function, UACR and HbA1c were given in the references [22, 23]. The Chronic Kidney Disease Epidemiology Collaboration algorithm was used to calculate estimated glomerular filtration rate (eGFR) [24].

### Diagnosis of DKD

The diagnosis of DKD was established by assessing the loss in renal filtration function or the presence of higher levels of urine albumin. The parameters used to indicate a decline in renal filtration function and an increase in urine albumin levels were an eGFR < 60 ml/min/1.73 m^2^ and a UACR ≥ 30 mg/g [25].

### Statistical analysis

The data from the NHANES dataset were processed via R (version 4.2.2). Significant findings were defined as having a two-sided *P* value < 0.05. The individuals were categorized into four groupings according on the quartiles of their eating midpoint. Continuous variables that follow a normal distribution were presented using the mean ± standard deviation, while continuous variables that do not follow a normal distribution were presented using the median and the interquartile range (25th and 75th percentiles). The representation of categorical data was done via frequencies, expressed as percentages. The study examined the variations in normally distributed, nonnormally distributed, and categorical data among the four subgroups using the one-way analysis of variance (ANOVA), Kruskal‒Wallis, and chi-square tests, respectively. UACR values did not follow a normal distribution, and therefore were transformed using natural logarithms. Two multivariate linear regression analyses were conducted to examine the mean differences in eGFR and ln(UACR) among the four subgroups, using the first quartile (Q1) as the reference. Additionally, a binomial logistic regression analysis was performed to evaluate the odds ratios (ORs) for DKD among the four subgroups. Since age, sex, ethnicity, household income, BMI, smoking and drinking status, energy intake, HbA1c and so on were all clinical variables which might affect the incidence of DKD, they were adjusted as covariables multivariate linear regression analyses and binomial logistic regression analysis. Furthermore, the whole population was categorized into two groups based on the duration of eating window: those who had meals for less than 12 h and those who consumed meals for 12 h or more. Afterwards, a comparison was made between the two categories in terms of the disparities in eGFR, UACR, and DKD incidence. Finally, the ORs for DKD were examined in participants with an eating window of less than 12 h, as opposed to those with an eating window of 12 h or more.

## Results

### Clinical characteristics of the study participants

The clinical features of the whole population and the four subgroups, categorized by the quartiles of eating midpoint, are shown in Table 1. The research included 2194 diabetic individuals, with an average age of 60.83 ± 13.15 years. Among these, 46.4% had DKD. Significant variations were observed among the four subgroups in terms of age, male proportion, race, BMI, smoking status, UACR, DKD incidence, first meal time, last meal time, and eating duration (*P* = 0.001, < 0.001, < 0.001, < 0.001, 0.037, 0.023, 0.037, < 0.001, < 0.001 and < 0.001, respectively). However, no significant differences were found in HbA1c, household income, drinking status, eGFR, and energy intake (all *P* > 0.05).

**Table 1:** Clinical characteristics of the study participants

Variables	Total	Q1	Q2	Q3	Q4	P value
Eating midpoint (hours)	8.00-22.50	< 13.38	13.38–14.13	14.14-15.00	> 15.00	
*n*	2194	501	560	568	565	
Age (years)	60.83 ± 13.15	61.36 ± 12.22	62.43 ± 12.83	60.17 ± 13.33	59.45 ± 13.91	0.001
Male, *n* (%)	1213(55.3)	319(63.7)	315(56.2)	284(50.0)	295(52.2)	< 0.001
BMI (kg/m^2^)	32.99 ± 7.74	32.52 ± 7.21	32.40 ± 7.33	32.81 ± 7.78	34.16 ± 8.40	< 0.001
Ethnicity, *n* (%)						< 0.001
Non-Hispanic White	731(33.3)	192(38.3)	204(36.4)	177(31.2)	158(28.0)	
Non-Hispanic Black	552(25.2)	99(19.8)	117(20.9)	158(27.8)	178(31.5)	
Mexican American	389(17.7)	103(20.6)	111(19.8)	94(16.5)	81(14.3)	
Other Race	522(23.8)	107(21.4)	128(22.9)	139(24.5)	148(26.2)	
Household income	2.39 ± 1.54	2.45 ± 1.51	2.50 ± 1.61	2.36 ± 1.53	2.27 ± 1.52	0.106
Smoking status, *n* (%)	1112(50.7)	232(46.3)	276(49.3)	313(55.1)	291(51.5)	0.037
Drinking status, *n* (%)	974(44.4)	239(47.8)	251(44.8)	240(42.2)	244(43.2)	0.309
HbA1c (%)	7.66 ± 1.78	7.58 ± 1.74	7.56 ± 1.69	7.76 ± 1.88	7.73 ± 1.80	0.141
eGFR (ml/min/1.73m^2^)	78.95 ± 30.04	78.60 ± 29.34	79.56 ± 30.62	79.68 ± 30.44	77.94 ± 29.71	0.735
UACR (mg/g)	13.73(7.36–46.70)	12.82(7.27–34.40)	15.51(7.79–68.37)	12.50(7.03–41.31)	15.21(7.46–45.82)	0.023
DKD, *n* (%)	1019(46.4)	215(42.9)	278(49.6)	243(42.8)	283(50.1)	0.037
The first meal time (hours)	8.30 ± 1.98	6.51 ± 1.19	7.65 ± 1.17	8.46 ± 1.06	10.39 ± 1.95	< 0.001
The last meal time (hours)	20.09 ± 1.53	18.62 ± 1.40	19.77 ± 1.17	20.51 ± 1.04	21.28 ± 1.15	< 0.001
Eating duration (hours)	11.78 ± 2.35	12.11 ± 2.22	12.12 ± 2.30	12.05 ± 2.04	10.89 ± 2.58	< 0.001
Energy intake (kcal)	1966.72 ± 751.71	1994.51 ± 823.78	1938.56 ± 735.42	1988.79 ± 723.71	1947.79 ± 728.46	0.509

Normally distributed continuous values in the table are given as the mean ± SD, skewed distributed continuous values are given as the median (25 and 75% interquartiles), and categorical variables are given as frequency (percentage) All times in this study are expressed on a 24-hour scale and converted into hours BMI body mass index, HbA1c glycosylated hemoglobin, eGFR estimated glomerular filtration rate, UACR urinary albumin-to-creatinine ratio, DKD diabetic kidney disease

### Multivariate regression analysis of eGFR and UACR in the eating midpoint quartiles

As shown in Table 2, in unadjusted and adjusted models the mean differences (B) in eGFR of the participants of other eating midpoint quartiles versus Q1 were not significant (all *P* > 0.05). The non-linear association between eating midpoint and eGFR was seen in Supplementary Figure S1.

**Table 2:** Mean differences (B [95% CI]) in eGFR among the quartiles of eating midpoint

Eating midpoint quartiles	Model 0	P value	Model 1	P value	Model 2	P value
eGFR, ml/min/1.73m^2^						
Q1	0-reference	-	0-reference	-	0-reference	-
Q2	0.96(-2.67 to 4.59)	0.605	1.51(-1.71 to 4.72)	0.358	1.34(-1.88 to 4.56)	0.415
Q3	1.08(-2.53 to 4.69)	0.560	-0.23(-3.43 to 2.96)	0.886	-0.31(-3.53 to 2.91)	0.849
Q4	-0.66(-4.29 to 2.96)	0.720	-2.03(-5.24 to 1.19)	0.216	-2.05(-5.28 to 1.17)	0.212

Model 0: unadjusted model

Model 1: adjusted for age, sex, ethnicity, household income, BMI

Model 2: additionally adjusted for smoking status, drinking status, energy intake, HbA1c

Table 3 also showed that only the adjusted mean difference (B) in ln(UACR) of the participants in the second quartile (Q2) of eating midpoint versus Q1 was significant (*P* = 0.003), and the corresponding (B) were 0.29 mg/g (95% CI, 0.10 to 0.49). After gradually adjusting for possible clinical variables, the adjusted mean difference (B) in ln(UACR) of Q2 versus Q1 remained significant (*P* = 0.003). In the fully adjusted model, the corresponding (B) were 0.31 mg/g (95% CI, 0.11 to 0.52). The non-linear association between eating midpoint and ln(UACR) was seen in Supplementary Figure S2.

**Table 3:** Mean differences (B [95% CI]) in UACR among the quartiles of eating midpoint

Eating midpoint quartiles	Model 0	P value	Model 1	P value	Model 2	P value
Ln(UACR), mg/g						
Q1	0-reference	-	0-reference	-	0-reference	-
Q2	0.29(0.10 to 0.49)	0.003	0.27(0.06 to 0.47)	0.010	0.31(0.11 to 0.52)	0.003
Q3	0.10(-0.08 to 0.30)	0.301	0.12(-0.08 to 0.32)	0.243	0.16(-0.05 to 0.36)	0.135
Q4	0.12(-0.07 to 0.31)	0.228	0.10(-0.10 to 0.30)	0.330	0.10(-0.10 to 0.31)	0.329

Model 0: unadjusted model

Model 1: adjusted for age, sex, ethnicity, household income, BMI

Model 2: additionally adjusted for smoking status, drinking status, energy intake, HbA1c

### Multivariate analysis of factors influencing DKD according to the eating midpoint quartiles

According to the findings shown in Table 4, individuals in the second and fourth quartiles (Q4) had higher ORs for DKD compared to those in the first one (Q1). The OR for Q2 was 1.31 (95% CI, 1.03 to 1.67), while the OR for Q4 was 1.33 (95% CI, 1.05 to 1.70) (*P* = 0.028 and 0.019, respectively). After controlling for additional clinical factors, the ORs for DKD remained significant in the individuals within the Q2 and Q4 (*P* = 0.016 and 0.027, respectively). The OR for Q2 was 1.42 (95% CI, 1.07 to 1.90), while the OR for Q4 was 1.39 (95% CI, 1.04 to 1.85).

**Table 4:** ORs (95% CIs) of DKD according to the quartiles of eating midpoint

Eating midpoint quartiles	Model 0	P value	Model 1	P value	Model 2	P value
Q1	1-reference	-	1-reference	-	1-reference	-
Q2	1.31(1.03–1.67)	0.028	1.43(1.09–1.88)	0.010	1.42(1.07–1.90)	0.016
Q3	0.99(0.78–1.27)	0.965	1.10(0.84–1.44)	0.498	1.15(0.86–1.53)	0.355
Q4	1.33(1.05–1.70)	0.019	1.44(1.10–1.89)	0.009	1.39(1.04–1.85)	0.027

Model 0: unadjusted model

Model 1: adjusted for age, sex, ethnicity, household income, BMI

Model 2: additionally adjusted for smoking status, drinking status, energy intake, HbA1c

### Association between eating duration and incidence of DKD

In Supplementary Table S3, no significant differences were seen between individuals with an eating duration of less than 12 h and those with an eating window of 12 h or more in terms of eGFR, UACR, and the incidence of DKD (all *P* > 0.05). Besides, when comparing individuals with an eating duration of less than 12 h to those with an eating duration of 12 h or more, the ORs for DKD were not significant, regardless of whether or not adjustment was used (all *P* > 0.05) (Supplementary Table S4).

## Discussion

The results of the current research indicate that a delayed eating midpoint may independently lead to a higher likelihood of developing DKD. Furthermore, the present investigation could not identify a distinct correlation between the duration of eating window and DKD. Moreover, it can be suggested that restricting meals to earlier timepoints throughout the daytime might potentially have positive effects on renal outcomes in individuals with diabetes.

Previous studies have proven the role of CR in improving diabetic renal outcomes. CR has the capacity to play a positive role in enhancing autophagy, diminishing inflammation and oxidative stress, and enhancing insulin sensitivity [26]. In rats with diabetes and obese individuals with type 2 diabetes, CR intervention can significantly reduce kidney damage [27, 28]. The aforementioned studies highlight the significance of CR in the context of diabetes and DKD. Nevertheless, the findings of this research demonstrated a significant correlation between the eating midpoint, irrespective of caloric intake, and the prevalence of DKD. Therefore, it may be necessary to direct focus not alone towards daily energy consumption, but also towards the eating midpoint.

DKD is a disease involving multiple factors, including abnormal glucose and lipid metabolism, inflammation, oxidative stress, abnormity in hemodynamics and so on [29]. In the present study, patients with a later eating midpoint tended to have a higher BMI. A later eating midpoint was associated with a later first meal time and last meal time. Clinical studies have confirmed the adverse effects of late meals [14, 15], and similar results were also observed in animal studies. Delaying the first meal of the day significantly increased body weight, fat deposition, and even affected the circadian rhythm of lipid metabolism associated genes in animals [30, 31]. In mice, late-night eating also led to weight gain, increased inflammation levels, and alternations in peripheral clock rhythms [32–34]. Therefore, eating late may influence the renal prognosis of patients with diabetes via multiple pathways, and adjusting the timing of meals may exert multiple benefits. A randomized crossover investigation conducted on males who were at risk of developing diabetes revealed a noteworthy reduction in the average blood glucose levels among participants who underwent the eTRE intervention, as opposed to those who received the delayed time-restricted eating (dTRE) intervention [35]. In addition, a group of healthy male participants underwent a 2-week eTRE intervention, which resulted in a significant decrease in systemic insulin sensitivity even ad libitum intake was permitted [36].

The benefits of eating early in the day may involve a variety of hormones and metabolic enzymes. Fibroblast growth factor-21 (FGF-21) and adiponectin are important regulators of energy metabolism, and the secretion of FGF-21 and adiponectin mostly occurs throughout 8 AM to 4 PM [37]. This temporal pattern of secretion is associated with the promotion of fat oxidation and glycolysis, while concurrently preventing fat formation [37]. Hence, it may be inferred that consumption of food during this period enhances the efficiency of nutrient digestion and metabolism in humans [38, 39]. Furthermore, it was observed that consuming the identical meal in the morning resulted in a greater glucose tolerance as compared to its consumption in the evening [40]. The phenomenon of nocturnal feeding is associated with a diminished responsiveness of the body to insulin, perhaps leading to oscillations in glucose levels [41]. In addition, due to the strong diurnal pattern of liver hydroxymethylglutaryl coenzyme A (HMG-CoA) reductase expression and cholesterol synthesis [42], eating earlier in the day may play a role in regulating lipid metabolism. Thus, having an early eating midpoint may be beneficial in the kidney by improving glycolipid metabolism.

Alireza and his colleagues conducted two trials of dietary interventions in treating experimental acute kidney injury (AKI), and found that restricting feeding for 5 h had renoprotective effects to rats with AKI by inhibiting the apoptosis of kidney cells, improving antioxidant status, and inhibiting renal fibrosis [43, 44]. Likewise, receiving short-term early time-restricted feeding could also exert the reno-protective effects of anti-oxidation and inhibition of renal fibrosis to rats by regulating mitochondrial dynamics [45]. Additionally, early time-restricted feeding could regulate the local immunity in the kidney of mice with hypertension [46]. In patients with CKD, eTRE intervention significantly improved the renal function, which might be attributed to alternations in gut microbiota [47]. These results provided several explanations in mechanisms for the results of this study.

Eating late in the day may contribute to circadian rhythm disruption as described previously. The circadian clock genes are not only present in the central nervous system, but they are also widely expressed in the kidney [6]. Research has shown the existence of a diurnal rhythm in both glomerular filtration function and urine protein excretion [48, 49]. Similarly, this study revealed that an earlier eating midpoint was possibly associated with less urinary protein excretion. Furthermore, the aberrant expression of clock genes may also expedite renal damage by several mechanisms, including elevated blood pressure, exacerbated inflammation, heightened oxidative stress, and intensified hypoxia [50]. Hence, eating early in the day has significant potential in enhancing renal outcomes in individuals with diabetes.

The challenge of dietary modification is that it possibly increases hunger of the subjects, making it difficult to sustain dietary modification. It is noteworthy that those who were subjected to the eTRE intervention exhibited a significant reduction in their appetite [51, 52]. Therefore, eating earlier in the day may be acceptable to patients with diabetes.

This study observed that the association between a long duration of eating window and the incidence of DKD trended towards statistical significance. Similarly, Nayara et al. also found that in elderly, the adults with a longer eating window had a non-significant lower prevalence of obesity and abdominal obesity, which was opposite to the results of non-elderly [20]. The underlying mechanism is that a short duration of eating window may be partly attributable to skipping breakfast, while ample evidence has confirmed the adverse effects of skipping breakfast in several metabolic morbidities [53–56]. In Korean middle-aged and older adults, skipping breakfast was significantly associated with increased risk of CKD [57].

The current research has provided novel insights by demonstrating that implementing time-restricted meals throughout the early part of the day may provide favorable kidney outcomes in diabetic individuals. Nevertheless, it is important to acknowledge certain limitations inherent in this research. The causal link between the two variables cannot be completely determined owing to the inherent limitations of the cross-sectional research design. Nor can it be ruled out that the relationship is in the opposite direction, specifically that subjects with DKD or other diabetic complications may alter their eating habits. Furthermore, the diagnostic criteria used in this investigation to identify DKD may introduce the possibility of confounding with other forms of CKD. Thirdly, in accordance with the eating midpoint algorithm, an earlier eating midpoint might perhaps indicate an earlier and shorter eating window. However, it is essential to get more accurate indications to further validate this assertion.

## Conclusions

The findings of this research provide evidence to support the notion that a more advanced eating midpoint is correlated with a reduced likelihood of developing DKD. Clinically, eating early in the day may have the potential to provide positive effects on renal outcomes among patients with diabetes. Further longitudinal and interventional investigations are required to substantiate the findings of this research.

### Electronic supplementary material

Below is the link to the electronic supplementary material.

Supplementary Material 1

## Data Availability

No datasets were generated or analysed during the current study.

## Declarations

The authors of this study thank the participants of the NHANES and the NHANES staff.
