# Supplementary material for: A late eating midpoint is associated with increased risk of diabetic kidney disease: a cross-sectional study based on NHANES 2013–2020
Source: Nutr J. 2024 Mar 23;23:39. doi: 10.1186/s12937-024-00939-z (PMC10960429; doi:10.1186/s12937-024-00939-z)
Supplement: Supplementary file 1 — Supplementary Material 1 [file 12937_2024_939_MOESM1_ESM.docx]

**Supplementary Figure S1 Scatter plot for the correlation of eating midpoint with eGFR**


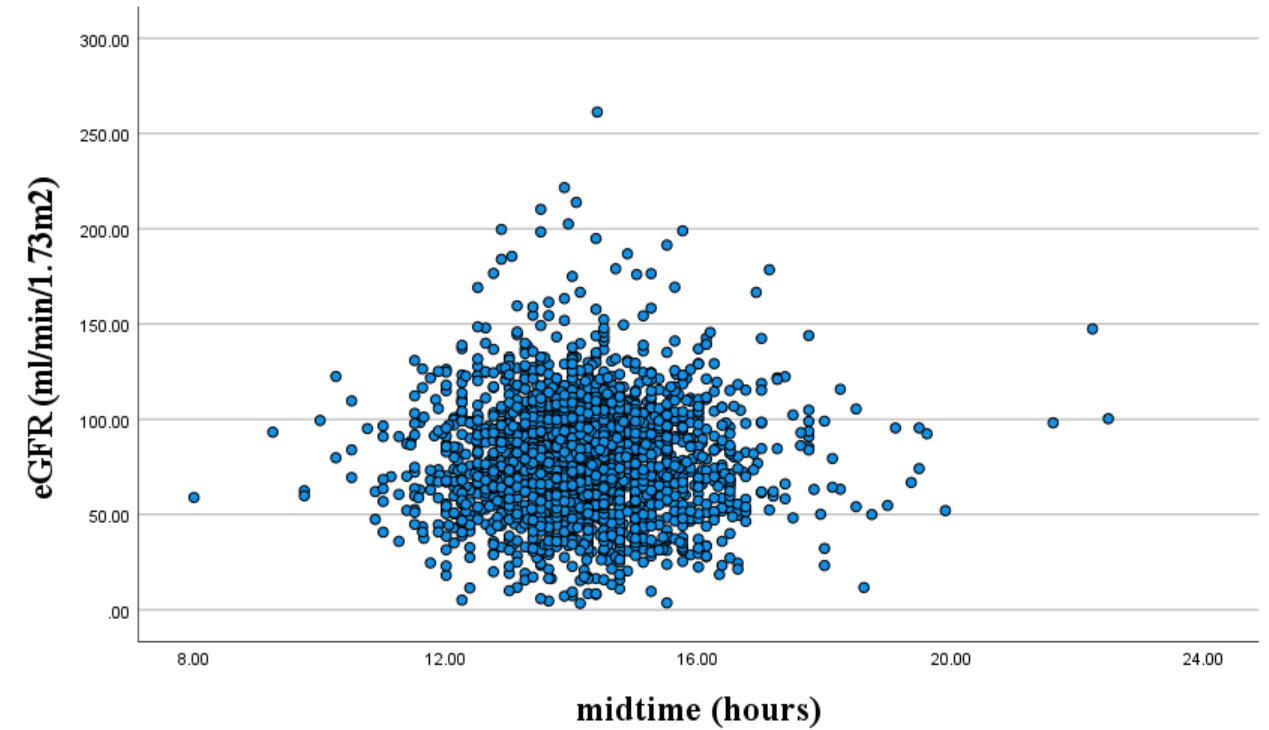


**Supplementary Figure S2 Scatter plot for the correlation of eating midpoint with ln(UACR)**


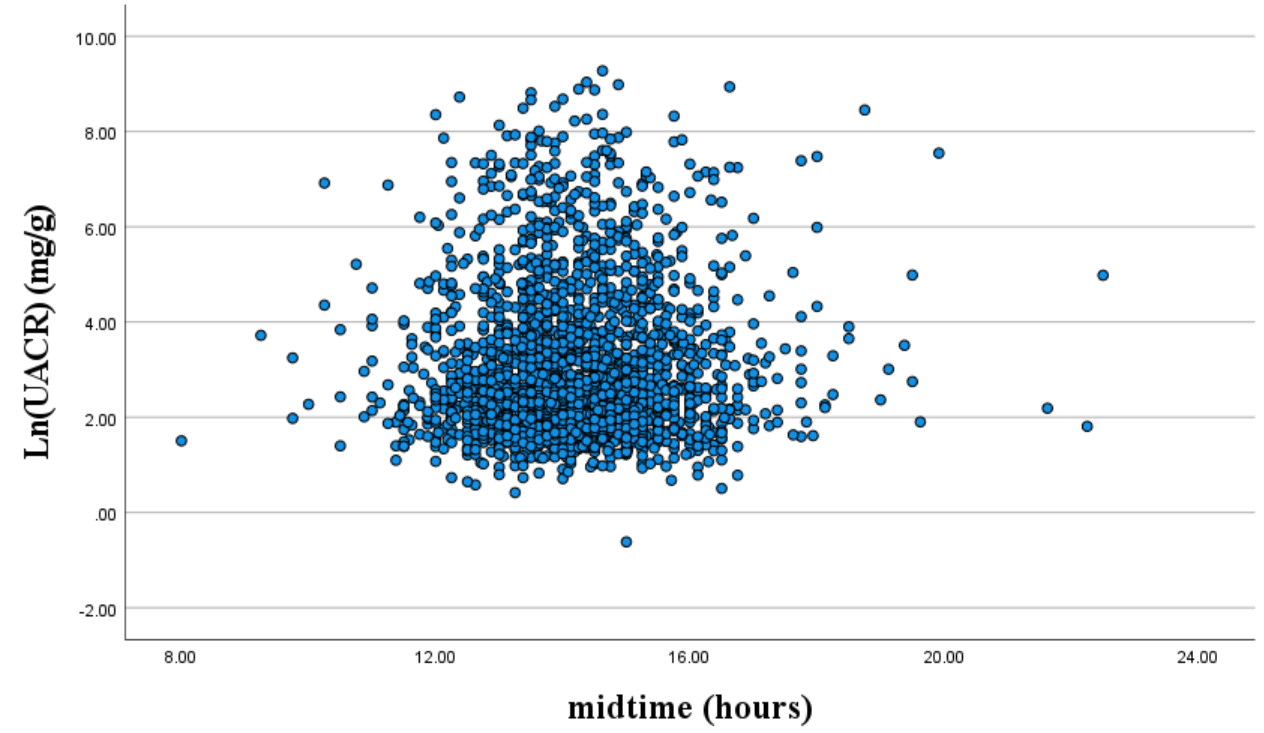


**Supplementary Table S1 Scatter plot for the correlation of eating midpoint with eGFR**

**Supplementary Table S2 Scatter plot for the correlation of eating midpoint with ln(UACR)**

**Supplementary Table S3 Clinical characteristics of the study participants**

| Variables | Total | Low eating duration | High eating duration | *P* value |
| --- | --- | --- | --- | --- |
| Eating duration (hours) | 11.78±2.35 | < 12 | ≥ 12 |  |
| *n* | 2194 | 1084 | 1110 |  |
| eGFR (ml/min/1.73m^2^) | 78.95±30.04 | 78.15±31.08 | 79.74±28.97 | 0.216 |
| UACR (mg/g) | 13.73(7.36-46.70) | 14.05(7.50-49.71) | 13.18(7.18-43.91) | 0.218 |
| DKD, *n* (%) | 1019(46.4) | 526(48.5) | 493(44.4) | 0.059 |

Normally distributed values in the table are given as the mean ± SD, skewed distributed values are given as the median (25 and 75% interquartiles), and categorical variables are given as frequency (percentage)

All times in this study are expressed on a 24-hour scale and converted into hours

eGFR estimated glomerular filtration rate, UACR urinary albumin-to-creatinine ratio, DKD diabetic kidney disease

**Supplementary Table S4 ORs (95% CIs) of DKD according to the binary classifications of eating duration**

| Eating duration | Model 0 | *P* value | Model 1 | *P* value | Model 2 | *P* value |
| --- | --- | --- | --- | --- | --- | --- |
| < 12 hours | 1-reference | - | 1-reference | - | 1-reference | - |
| ≥ 12 hours | 0.85(0.72-1.00) | 0.054 | 0.87(0.71-1.05) | 0.137 | 0.85(0.70-1.05) | 0.126 |

Model 0: unadjusted model

Model 1: adjusted for age, sex, ethnicity, household income, BMI

Model 2: additionally adjusted for smoking status, drinking status, energy intake, HbA1c
